# Complex network behavior in epileptic patients treated with Vagus Nerve Stimulation (VNS): VNS responders exhibit a unique pattern, different from VNS non-responders and healthy controls

**DOI:** 10.3389/fnins.2025.1662068

**Published:** 2026-01-27

**Authors:** Irena Dolezalova, Jan Chladek, Michal Macek, Jan Chrastina, Tereza Škvařilova, Petra Burilova, Stepan Erben, Eva Zatloukalova, Milan Brazdil

**Affiliations:** 1 The First Department of Neurology, Brno Epilepsy Center, Member of ERN-Epicare, St. Anne's University Hospital and Faculty of Medicine, Masaryk University, Brno, Czechia; 2 International Clinical Research Center, St. Anne's University Hospital, Brno, Czechia; 3 Behavioral and Social Neuroscience Research Group, CEITEC-Central European Institute of Technology, Masaryk University, Brno, Czechia; 4 Institute of Scientific Instruments, Czech Academy of Sciences, Brno, Czechia; 5 Brno Epilepsy Center, Department of Neurosurgery, St. Anne's University Hospital and Masaryk University, Brno, Czechia; 6 Institute of Biostatistics and Analyses, Faculty of Medicine, Masaryk University, Brno, Czechia; 7 Department of Health Sciences, Faculty of Medicine, Masaryk University, Brno, Czechia

**Keywords:** drug-resistant epilepsy, entropy, healthy controls, relative power, Vagal nerve stimulation (VNS)

## Abstract

**Introduction:**

Vagus nerve stimulation (VNS) represents an alternative treatment option in drug-resistant epilepsy. VNS patients can be categorized as responders (R, ≥50% seizure reduction) or non-responders (NR, < 50% seizure reduction). We demonstrate that VNS responders and VNS non-responders differ in their electrophysiological characteristics based on pre-implantation EEG analysis, specifically evaluated using relative mean power (RPW) and various information Entropy estimators computed in both he frequency and time domains. Based on the RPW and the Entropy estimators, we define and analyze the Unique Characteristics (UCs) of the individual (R and NR) groups of epileptic patients as well as Common Characteristics (CCs) that differentiate epileptic patients from healthy controls (HCs).

**Methods:**

We investigated pre-implantation time series in 59 epileptic patients treated with VNS (24 VNS responders, 35 VNS non-responders). Subsequently, we acquired the EEG time series for 37 age- and gender-matched HCs. The EEG recordings of these three groups were filtered into standard frequency bands (theta, alpha, beta, and gamma) and segmented into eight consecutive time intervals, containing specific types of stimulation and resting states. For each of these segments, the RPW and seven Entropy estimators were calculated. We focused on the distribution of features differentiating between the epileptic patients (VNS responders or non-responders) and the HCs.

**Results:**

We identified 41 UCs (7 in RPW, 34 in Entropy) of VNS responders, in contrast to 19 UCs (4 in RPW, 15 in Entropy) of VNS non-responders. The UCs of VNS responders exhibit a specific pattern, showing their binding in the frequency domain to the alpha band and temporal binding to the segments of hyperventilation stimulation. The UCs of VNS non-responders were also temporally linked to hyperventilation, but mainly in the theta and gamma frequency bands.

**Conclusion:**

The VNS responders exhibit more differences when compared to HCs than VNS non-responders. These differences can be observed in RPW, but they become more pronounced when Entropy analysis is applied. It seems that the distinct response to hyperventilation is present in both VNS responders and non-responders, differentiating them from HCs. However, the binding of this response to frequency bands differs among VNS responders and non-responders. In particular, the reaction among the VNS responders is strongly associated with the alpha frequency band.

## 1 Introduction

Epilepsy is a disease characterized by epileptic seizures. In most patients, treatment with anti-seizure medication (ASM) leads to complete seizure freedom. Approximately one-third of epileptic patients continue to experience seizures despite adequate ASM therapy. These drug-resistant patients can be candidates for resective brain surgery, which is the only method offering long-term and sustained seizure freedom for drug-resistant epilepsy patients (Kwan et al., 2010; Engel, 2018). However, not all epileptic patients can undergo brain surgery for various reasons (e.g., the localization or the extension of an epileptogenic zone or a generalized form of epilepsy). In cases where brain resection is not applicable, neurostimulation appears to be a suitable alternative (Simpson et al., 2022).

Vagus nerve stimulation (VNS) is the most widely used form of neurostimulation. VNS, as well as other neurostimulation techniques, only rarely leads to complete seizure cessation. VNS efficacy is traditionally rated in terms of the percentage of seizure reduction. Patients who benefit from VNS (≥50% seizure reduction) are labeled as VNS responders. Patients who have limited or no benefit from this therapy (< 50% seizure reduction) are called VNS non-responders (McHugh et al., 2007).

It appears that the VNS response can be assessed pre-implantation by analyzing specific patient biomarkers (Workewych et al., 2020). The response can be estimated or predicted using several methods, including magnetic resonance imaging (MRI) and electroencephalography (EEG) post-processing (Workewych et al., 2020). Recently, we focused on the pre-implantation predictors of VNS efficacy in patients with epilepsy, distinguishing between VNS responders and non-responders based on pre-implantation EEG recordings. We analyzed two features for the time series, the relative mean power (RPW) (Brázdil et al., 2019) and the information Entropy (Sklenarova et al., 2023) of the EEG signal. While the RPW roughly reflects the overall activity in the parts of the brain nearest to each electrode, the Entropy measures the complexity of this activity. It thus provides a more nuanced indicator of its various changes. We estimated the Entropy by several different algorithms in a frequency (Spectral Entropy) or in a time domain (Approximate Entropy, Sample Entropy, Empirical Permutation Entropy for Ordinal Patterns, Empirical Permutation Entropy for Ordinal Patterns with Tied Ranks, Robust Empirical Permutation Entropy, and Empirical Conditional Entropy of Ordinal Patterns) (Sklenarova et al., 2023).

We selected these two features, RPW and the Entropy estimators, because of their relation to the mechanism of presumed VNS action. Despite some ambiguities, VNS appears to act by desynchronizing brain circuits (Chase et al., 1967; Sangare et al., 2020). This desynchronization can be an opposing mechanism to the pathological synchronization that is seen during epileptic seizures (Chase et al., 1967). We demonstrated that some individuals can react with more pronounced desynchronization to external stimuli than others; this ability is likely tightly linked to VNS efficacy and is reflected in RPW (Brázdil et al., 2019; Koritáková et al., 2021; Sklenarova et al., 2023; Jurková et al., 2024).

The degree of synchronized and desynchronized brain states, characterized by different levels of predictability and regularity, can have significant implications for VNS treatment. Synchronized states (more regular and predictable) might respond differently to VNS compared to desynchronized states (less regular and predictable). All these characteristics can be reflected in Entropy-based biomarkers, which, together with the RPW, allowed us to distinguish between groups of VNS responders and non-responders (Brázdil et al., 2019; Sklenarova et al., 2023). However, we are still missing one crucial piece of information: it is unclear what type of response to VNS (responders vs. non-responders) is closer to the general healthy population. This task is addressed in the current study, in which we aim to identify whether and in which aspects the neuronal network behavior of VNS responders or VNS non-responders more closely resembles that of the general healthy population (represented here by the HC group), when performing RPW and Entropy analysis. This information is crucial when trying to understand the mechanisms of VNS action, which is yet to be fully understood (Gargus et al., 2024).

## 2 Methods

**Figure 1:**
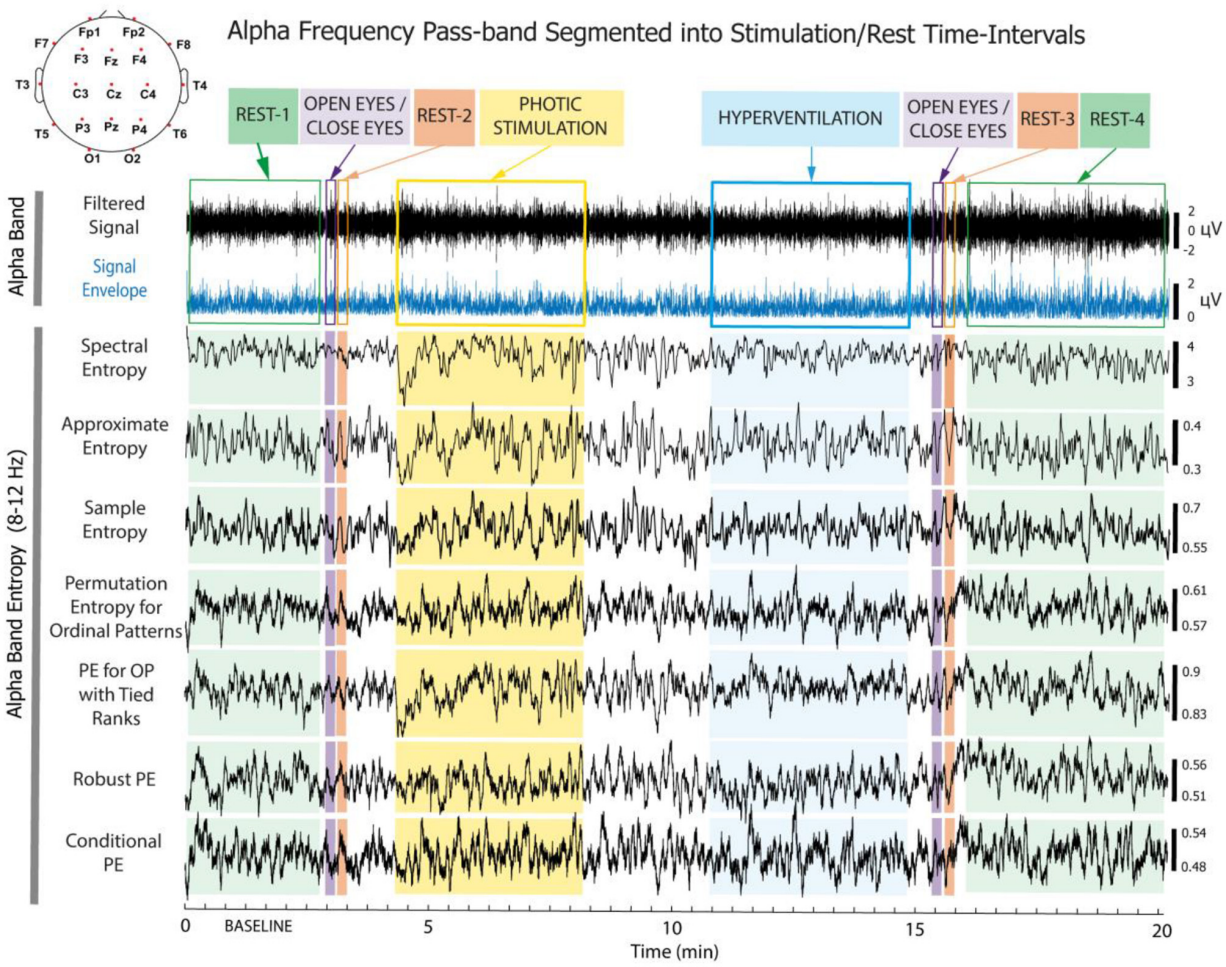
The methodology of EEG data pre-processing. The predefined measurement protocol contained the following eight time intervals: (1) Rest-1 (baseline), (2) eyes opening/closing-1 (OC-1), (3) Rest-2, (4) photic stimulation (PS), (5) hyperventilation (HV), (6) OC-2, (7) Rest-3, and (8) Rest-4. The displayed single-healthy-subject EEG time series recorded from electrode C3 and precomputed in the alpha band (8-12 Hz) represents the filtered signal, the signal envelope calculated using the Hilbert transform, and differences in the Spectral Entropy, Approximate Entropy, Sample Entropy, Empirical Permutation Entropy and Ordinal Patterns Distributions, Empirical Permutation Entropy with Tied Ranks, Robust Empirical Permutation Entropy, and Conditional Permutation Entropy of Ordinal Patterns estimated from the same signal using a 4s moving window.

In the current study, we focus on the differences in RPW and Entropy based on EEG analysis in drug-resistant epileptic patients treated with VNS and in healthy controls (HCs). We identified a group of adult epileptic patients implanted with VNS. Based on their clinical responses to VNS, the epileptic patients were divided into two groups: VNS responders and VNS non-responders based on their 2 year postoperative outcome. We subsequently identified age- and gender-matched HCs as the third group. In all three groups (both epileptic patient groups and HCs), we acquired EEG recordings based on a predefined protocol (EEG containing specified time intervals with photic stimulation and hyperventilation in a given order). In all the epileptic patients, the EEG was recorded before VNS implantation (pre-implantation EEG was filtered into standard frequency bands and segmented into eight time intervals, demarcated by different means of stimulation and rest states (see Figure 1). The EEG recordings were then processed in terms of RPW (Brázdil et al., 2019; Koritáková et al., 2021; Dolezalova et al., 2022), and was calculated using seven different algorithms. Further analyses concerned the differences between VNS responders vs. HCs and VNS non-responders vs. HCs.

The Ethics Committee of St. Anne's University Hospital approved the study. All participants provided informed consent for the use of their data.

### 2.1 Subject selection criteria

#### 2.1.1 Epileptic patient selection

We retrospectively included patients treated for drug-resistant epilepsy for whom VNS was indicated based on a clinical decision after a comprehensive evaluation of their clinical data. All patients underwent thorough investigation before VNS implantation.

The criteria for the epileptic patient inclusion in the study were: (1) drug-resistant epilepsy, (2) availability of good-quality pre-implantation on a defined protocol (the protocol is described in detail in the following paragraph), (3) the determined VNS efficacy in terms of VNS responders (≥50% seizure reduction) and VNS non-responders (< 50 % seizure reduction) at the second year after the stimulation initiation based on the McHugh criteria (McHugh et al., 2007). We excluded patients without good-quality EEG recordings or with undetermined VNS efficacy.

#### 2.1.2 Healthy controls selection

We included age- and gender-matched HCs, in whom the EEG was recorded based on a predefined protocol. The HCs were individuals without a history of brain disease, with no intake of drugs influencing the central nervous system (CNS; namely anti-depressant, antipsychotics, or benzodiazepines), with no history of substance abuse, and with normal MRI scans.

### 2.2 Data Acquisition-EEG recording

EEGs were recorded at a sampling rate of 128 Hz on an Alien Deymed system with electrodes placed on the head according to the standardized international 10-20 system. We analyzed *n* = 19 electrodes with a reference electrode placed on the mastoid, as schematically depicted in Figure 1. Standard antialiasing filters were used before digitalization. The occasional artifacts were excluded from the analysis semi-automatically using the FieldTrip Toolbox Oostenveld et al., (2011) and then manually checked.

We included only participants with EEG recorded based on a predefined protocol performed with eyes closed (except for specified opening intervals), comprising the following 8 time intervals: (1) Rest-1 (baseline; ~2 min), (2) eyes shortly opened and closed (OC-1; 10 s), (3) Rest-2 (immediately after eye closure; 10 s), (4) photic stimulation (PS; ~4 min), (5) hyperventilation (HV; ~4 min), (6) eyes shortly opened and closed (OC-2; 10 s), (7) Rest-3 (immediately after eye closure; 10 s), and (8) Rest-4 (~2 min) (see Figure 1). Considering the photic stimulation, the specific protocol involved changing the frequencies in trains of about 10 s for each frequency, with eyes closed. The frequency sequence proceded as (5, 10, 15, 20, 25, 30, 40, 50, 40, 30, 20, 15, 10, 5) Hz.

The EEG signals recorded from 19 electrodes were filtered into four standard frequency bands (theta 4-7.5 Hz, alpha 8-12 Hz, beta 14-30 Hz, and gamma 31-45 Hz). The filtering was performed using a two-pass (zero-phase) Butterworth IIR filter implemented in FieldTrip (Oostenveld et al., 2011). The filter order was set to 4, resulting in-6 dB attenuation at the cut-off frequencies.

We define the time-frequency segment as a primary unit of analysis, yielding a total of 32 distinct segments per electrode (derived from 8 time intervals × 4 frequency bands). Subsequently, from each segment, a set of 8 quantitative features (1 RPW value and 7 distinct Entropy estimators) was extracted and normalized against the initial Rest-1 interval. In total, we analyzed 19 x 32 x 7 = 4256 normalized feature values, respectively 4864 values including the baseline.

### 2.3 Data processing

#### 2.3.1 Relative mean power (RPW)

Relative mean power was analyzed in the same manner as described in our previous article (Brazdil 2019). First, absolute mean power was computed as the mean value of the power envelope evaluated in the time domain using the Hilbert transform for predefined frequency passbands. We note that the alternative frequency-domain-based Welch method for calculating RPW provides comparable results, and we chose the Hilbert method, as it is typically preferrable for non-stationary data, which is often the case in our EEG signals (especially in the time intervals during the stimulations).

Subsequently, RPWs were calculated as the percentage decrease or increase in the average power value at each time interval within a given frequency band, for each electrode, and relative to the baseline value. We then compared the RPW values between the VNS responders and the HC group, as well as between the VNS non-responders and the HC group, individually for each electrode and EEG segment characterized by time intervals and frequency bands.

#### 2.3.2 Entropy

Entropy is a fundamental concept in information theory, introduced by Shannon (1948). Shannon's equation


$$\begin{array}{l} H=-\overset{n}{\underset{i=1}{\sum }}p_{i}log\left(p_{i}\right) \end{array}$$

Involves specific probability distributions p_i_ of a suitable random variable X, attaining values X_i_ with probability p_i_. X can be any quantity that characterizes the system dynamics in some practical way. The dynamics of the network of neurons in the brain can be mathematically seen as a dynamical system evolving on an attractor (in a steady state) or transitioning between different attractors, when external stimulation or internal conditions change. Entropy can be a suitable quantifier reflecting the properties of such attractors (Unakafova and Keller, 2013; Datseris et al., 2023) and also aims to measure the degree of uncertainty (lack of information) related to the dynamics. Alternatively, the higher the entropy value, the greater the degree of irregularity and complexity in the system. However, estimating Entropy accurately from time-series data in clinical setting is not a simple task; this has led to the development of numerous variants and algorithms for entropy estimation, each based on different postulates of the probability distribution function. It has been recognized that different entropy estimates, used in conjunction with empirically determined computational parameters, can provide complementary information (Anier et al., 2012; Amarantidis and Abásolo, 2019). Based on these findings, several comprehensive reviews (Pincus, 1991; Alcaraz et al., 2010; Liang et al., 2015; Traversaro et al., 2018; Delgado-Bonal and Marshak, 2019; Zanin and Papo, 2021; Amigó et al., 2022; Frohlich et al., 2022; Lau et al., 2022), and previous work of Sangare et al. (2020), we evaluate seven different estimates of Entropy in this study in the spectral and time domains that are computed from a time series generated by a complex dynamic system during different conditions (Table 1):

**Table 1:** The characteristics of individual Entropy methods.

**1)**	***Spectral Entropy***
	Spectral Entropy measures the regularity of a signal in the frequency domain. A normalized power distribution can be viewed as a probability distribution, and then Spectral Entropy can be estimated as Shannon's Entropy Shannon, (1948). It was first used to measure the irregularity of α-rhythm EEG by Inouye et al. (1991), who used the in-band Entropy in four main frequency bands. The disadvantage of spectrum-based methods is that they reflect only the linear characteristics of the system (Jordan et al., 2008), unlike the estimators of the Kolmogorov-Sinai entropy discussed below.
	We used a MATLAB implementation of the normalized power spectral density estimate via Welch's method (SpectEn), with a 4 s moving window.
**2)**	***Approximate Entropy***
	Approximate Entropy, introduced by Pincus (1991) (as are all other methods discussed below), is determined in the time domain and approximates the fundamental Kolmogorov-Sinai Entropy of state space attractors, including their non-linear characteristics involving the exponential divergence/convergence of trajectories on the attractors in the phase space (Unakafova and Keller, 2013). The Approximate Entropy approach is based on sequence repetition and recurrence rather than time-series data distribution (Lee et al., 2013). The method was designed to measure the randomness of noisy data and to distinguish between two groups of time series. It has been used in heart rate analysis to differentiate between healthy and sick newborns (Pincus, 1991)
	To calculate Approximate Entropy (AppEn), we applied an algorithm implemented in MATLAB with the following parameters: a 4 s moving window, an embedding dimension of 2, and a time delay of 1 point.
**3)**	***Sample Entropy***
	Sample Entropy overcomes some disadvantages of Approximate Entropy–specifically its bias, relative inconsistency, and strong dependence on data length (Richman and Moorman, 2000). Sample Entropy avoids counting self-matches and therefore reduces its bias. It has three empirical parameters crucial for estimating entropy values. However, their optimal setting is unclear; this should be the subject of further research (Richman and Moorman, 2000; Liang et al., 2015; Chen, 2019; Xiong et al., 2019).
	We used the Sample Entropy (SampEn) function based on work of Richman and Moorman (2000) with a 4 s sliding time window, embedding dimension 2, tolerance 0.1 of standard deviation, and Euclidean distance type.
**4)**	***Empirical Permutation Entropy for Ordinal Patterns***
	Permutation Entropy, introduced by Bandt and Pompe (2002) and extended by Unakafova (2015) and Keller et al. (2014), has been proposed to measure the complexity of general high-dimensional time series in the real world without restricting the process of their generation (Jordan et al., 2008), i.e., it is practically suitable for regular, chaotic, or noisy systems. It is based on a combination of the concept of symbolic dynamics and Entropy. This algorithm maps a time series for ordinal patterns, a set of not necessarily overlapping permutation patterns. Empirical Permutation Entropy is a variant of permutation entropy that takes into account the empirical probabilities of the ordinal patterns.
	In our particular case, we used the opdPE method implemented in Unakafova (2018), with a window size of 4 s, embedding dimension 3, ordinal patterns of order 6, and time delay 1 between points in ordinal patterns.
**5)**	***Empirical Permutation Entropy for Ordinal Patterns with Tied Ranks***
	To avoid possible misclassification due to bias resulting from high permutation order and violations of the condition that the data do not contain the same consecutive values (Jordan et al., 2008), we also used the Empirical Permutation Entropy for Ordinal Patterns with Tied Ranks. This method is an improvement of the Empirical Permutation Entropy for ordinal patterns, so that the method is adapted to the same values of the “tied ranks” that can occur with high frequency in the time series (Unakafova, 2015). The limitation is in the number of precomputed lookup tables for commonly used embedding dimensions.
	We used the PEeq method, adopted from Unakafova (2018) with the following parameters: window size of 4 s, ordinal patterns order 3, and delay 3 between points in ordinal patterns.
**6)**	***Robust Empirical Permutation Entropy***
	Robust Permutation Entropy was proposed in Unakafova (2015) and Keller et al. (2014). The method increases the robustness of Permutation Entropy to noise and abnormal changes such as artifacts. It counts only the “robust” ordinal patterns with a sufficient number of reliable point pairs. The disadvantage is that it depends on the setting of its parameters, such as thresholds.
	We used the RePE method implemented in Unakafova (2018) with the following parameters: window size of 4 s, ordinal pattern of order 6, delay 1 between ordinal pattern points, lower threshold 0.2, and upper threshold 100
**7**	***Conditional Entropy***
	The algorithm for calculating Empirical Conditional Entropy of Ordinal Patterns was proposed in 2014 Unakafov and Keller (2014). While permutation entropy characterizes the variety of ordinal patterns themselves, conditional Entropy characterizes the average variety of patterns that follow a given ordinal pattern (Porta et al., 1998; Zanin and Papo, 2021).
	We used an algorithm based on corrected conditional entropy, implemented as CE with the parEntropy parameters: a window size of 4 s, an ordinal pattern order of 3, and a delay of 1 between points in ordinal patterns (Unakafov and Keller, 2014).

### 2.4 Statistics

#### 2.4.1 Statistics for demographics

Demographic data were compared in two ways: (1) Representation of males and females in the three groups of subjects was tested using the Freeman-Halton extension of Fisher's exact test and (2) and a generalized linear regression model (GLM) with binomial distribution and logit function was used to examine the relationship between patient response to treatment and confounding demographic and medication factors. The GLM model was tested for collinearity of variables using the inflation variance factor (IVF).

#### 2.4.2 Statistics for signal characteristics

Comparisons of the RPW and all the Entropy signal characteristics among the three different subject groups were performed using the Kruskal-Wallis test, followed by a Dunn-Sidak correction. Since the values obtained from the scalp electrodes are not independent, *p*-values for all 19 electrodes were corrected for multiple comparisons in each time interval and frequency band using Combined Fisher Probability (CFP) correction. CFP correction is reported as an alternative to the FDR or Holm-Bonferroni correction, which can be considered too conservative (Whitlock, 2005; Diz et al., 2011). For CFP, we used the MATLAB Multiple Testing Toolbox (Martínez-Cagigal et al., 2025). After applying the multiple comparison correction to the 19 *p*-values, the corresponding number of h-values can be used for each time interval and frequency band as a “logical variable” value, distinguishing between zero and non-zero numbers of significant electrodes in each of the total of *N* = 32 segments. This approach subsequently enables us to define both unique and common characteristics, and to evaluate their counts (CC or UC). All statistical comparisons were assessed at the conventional alpha level of 0.05.

### 2.5 Unique and common characteristics for R and NR subject groups

To compare and classify the distinct responses of the R and NR epileptic patient subjects concerning the HC subjects, we introduce here the following threefold distinction, applied separately for each of the RPW and the Entropy estimators:

1) **Unique characteristics of VNS responders (UCsR)** are defined as differences present only between VNS responders and HC in a given feature (RPW, Entropy estimators) in the individual EEG segments. In other words, the number N of UCsR is provided by the number of segments, which display *a non-zero number* of significant electrodes exclusively in the R vs. HC and not the NR vs. HC groups;
2) **Unique characteristics of VNS non-responders (UCsNR)**are defined in analogy with the previous, as differences present only between VNS non-responders and HC in a given feature (RPW, Entropy) in the individual EEG segments;
3) **Common characteristics of VNS responders and non-responders (CCs)** are defined as differences present concomitantly in VNS responders and VNS non-responders, differentiating the epileptic patients from the HCs in a given feature in a defined EEG segment. Thus, the number N of CCs is provided by the number of segments that display *any non-zero number* of significant electrodes for both the R vs. HC and NR vs. HC groups. We expect the CC characteristics to be more closely related to epilepsy or epilepsy treatment than to the efficacy of VNS.

## 3 Results

### 3.1 Demographic data

We identified a group of 59 epileptic patients treated with VNS−35 (59%) responders and 24 (41%) non-responders. There were 34 (58%) women and 25 (42%) men. The median age at epilepsy development was 9 years (min-max 1-51 years), and the median duration of epilepsy at VNS implantation was 22 years (min-max 4-60 years). The median age at the time of EEG recording was 32 years (min-max 18-65 years). The epilepsy was characterized as focal in 56 (95%) subjects, and as generalized in 3 (5%) subjects.

The HC group consisted of a total of 37 enrolled subjects, comprising 18 women (49%) and 19 men (51%). The median subject age during EEG recording was 39 years (min-max 16-77 years).

Mutual comparison between the R and NR epileptic patient groups using a Generalized Linear Model (GLM) results in no significant predictors (Age at epilepsy onset: *p*-value = 0.076, Age at VNS implantation: *p*-value = 0.070, Gender at the limit of significance: *p*-value = 0.0504, EEG measurement time before VNS implantation: *p*-value = 0.167, and type of epilepsy: *p*-value ≥0.485. Variance Inflation Factor (VIF) ≤ 3.169, mean VIF = 1.672).

Comparison of the R and NR groups of epileptic patients with healthy controls (HCs) using the Kruskal-Wallis test similarly does not find any significant differences concerning age, with χ^2^(2, *N* = 96) = 4.64, *p* = 0.0985. Regarding the sex distribution across the groups, the Freeman-Halton extension of the Fisher exact probability test again reveals no significant differences in age among the three groups, with χ^2^(2, *N* = 96) = 3.58, *p* = 0.167.

All statistical comparisons were assessed at the conventional alpha level of 0.05.

### 3.2 Unique and Common Characteristics in RPW analysis

**Figure 2:**
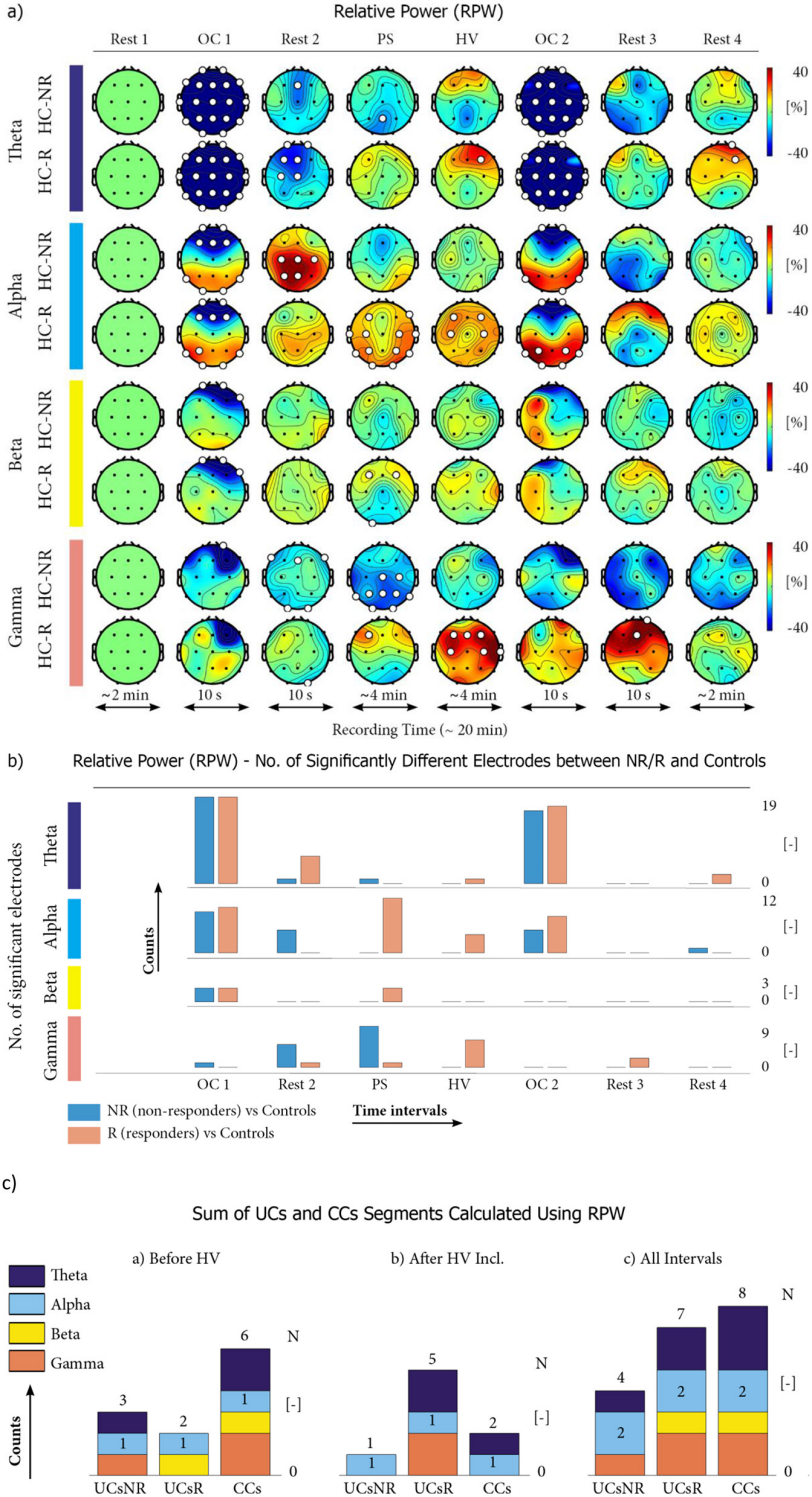
Relative power (RPW)–the differences between healthy controls (HCs) and VNS responders (R) or VNS non-responders (NR). **(a)** Distributions of the RPW over the scalps for eight consecutive time intervals (Rest-1, OC-1, Rest-2, PS, HV, OC-2, Rest-3, and Rest-4) indicating the electrodes with statistically significant (white circles) and insignificant (black dots) differences (at the *p* ≤ 0.05 level) between VNS responders vs. HCs and VNS non-responders vs. HCs (shown in pairs of separate lines below one another) across individual frequency bands (theta, alpha, beta, and gamma). Each “head” represents the RPW in a specific EEG segment defined by frequency (rows) and time interval (columns), with RPW values calculated separately for each scalp electrode. **(b)** Time distribution of EEG segments with numbers *n* of electrodes showing statistically significant differences in RPW between VNS responders (red columns) and VNS non-responders (blue columns) when compared to HCs. The high point of each column correlates with the number of electrodes with these significant differences. **(c)** The number of unique characteristics of non-responders (UCsNR) is compared to the number of unique characteristics of responders (UCsR) and the number of common characteristics of responders and non-responders (CCs) summed across all frequency bands obtained using RPW analysis. Three different temporal views are shown: **(a)** counts before hyperventilation (including photic stimulation), **(b)** counts after photic stimulation (including hyperventilation), and **(c)** counts during all time intervals

When analyzing the differences between epileptic patients (R and NR) and the healthy controls using RPW, we found statistically significant differences in N = 27 EEG segments (Figures 2a, b). When splitting this number to the R and NR, N = 15 segments were found for R and *N* = 12 segments for NR (Figure 2b; numbers of red or blue bars with non-zero height, respectively). Below, we specify which of these constitute unique characteristics (UC) for each individual R or NR group and which are common characteristics (CC). The distribution of UCs and CCs over the individual frequency bands and temporal segments is summarized in Figure 2c.

**UCs of VNS responders:** We identified *N* = 7 UCsR for VNS responders. They are present and relatively uniformly distributed among all frequency bands (*N* = 2 segments in theta, alpha, and gamma). Still, temporally, they predominate in the second part of the EEG recording, which includes the PS and HV stimulation (*N* = 2 segments in PS, *N* = 3 in HV, and *N* = 1 in Rest-3). This finding highlights the importance of the activation methods via PS and HV for VNS responders.

**UCs of VNS non-responders**: We identified *N* = 4 UCsNR of VNS non-responders; however, their distribution within the time intervals and frequency bands differed and seemed to be more random (only *N* = 1 UCsNR in all of OC-1, Rest-2, PS, and Rest-4).

**CCs of VNS responders and non-responders**: There were *N* = 8 CCs; most of them were bound to the episodes with eyes opening/closing (*N* = 3 CCs in OC-1, *N* = 2 CCs in OC-2) or proximity to eyes opening/closure (*N* = 2 CCs in Rest-2), predominantly in the theta and alpha frequency bands.

**Summary of the RPW analysis:** The VNS responders differ from HCs in the time intervals during which stimulation by PS and HV is applied. This special “VNS responders' pattern” is significantly less pronounced in VNS non-responders. On the other hand, an abnormal reaction to eyes opening was found among both the VNS responders and non-responders, and thus constitutes a CC, differentiating the epileptic patients from HCs. When focusing on the distribution of UCs and CCs among individual frequency bands in RPW, we do not find a marked dominance of any of the frequency bands.

### 3.3 Unique and common characteristics in entropy analysis

**Figure 3:**
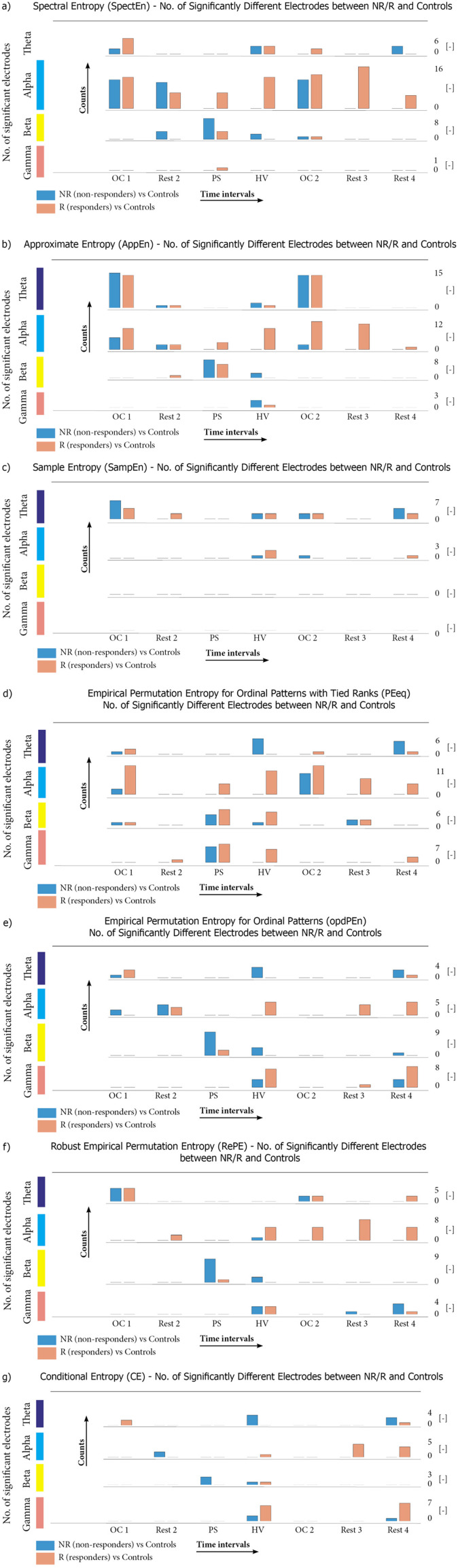
The differences between healthy controls (HCs) and VNS responders or VNS non-responders expressed by the individual Entropy estimators. Each panel **(a–g)** represents the differences in the individual frequency band (theta, alpha, beta, and gamma) for each Entropy estimator (analogous to Figure 2b). The significant differences between VNS responders and HCs are marked in red; the differences between VNS non-responders and HCs are marked in blue. The high point of the column corresponds to the number of electrodes, with a statistically significant difference.

All of the Entropy estimators display relatively large numbers *N* of EEG segments differentiating epileptic patients (R and NR) and HC controls, as can be seen in Figure 3. In order of decreasing N, the values are as follows: Empirical Permutation Entropy for Ordinal Patterns with Tied Ranks (*N* = 27, Figure 3d), Approximate Entropy (*N* = 24, Figure 3b) and Spectral Entropy (*N* = 23, Figure 3a) revealed the highest number of EEG segments. They were followed by Empirical Permutation Entropy for Ordinal Patterns (*N* = 20, Figure 3e), Robust Empirical Permutation Entropy (*N* = 19, Figure 3f), Conditional Entropy (*N* = 15, Figure 3g), and Sample Entropy (*N* = 13, Figure 3c).

**Figure 4:**
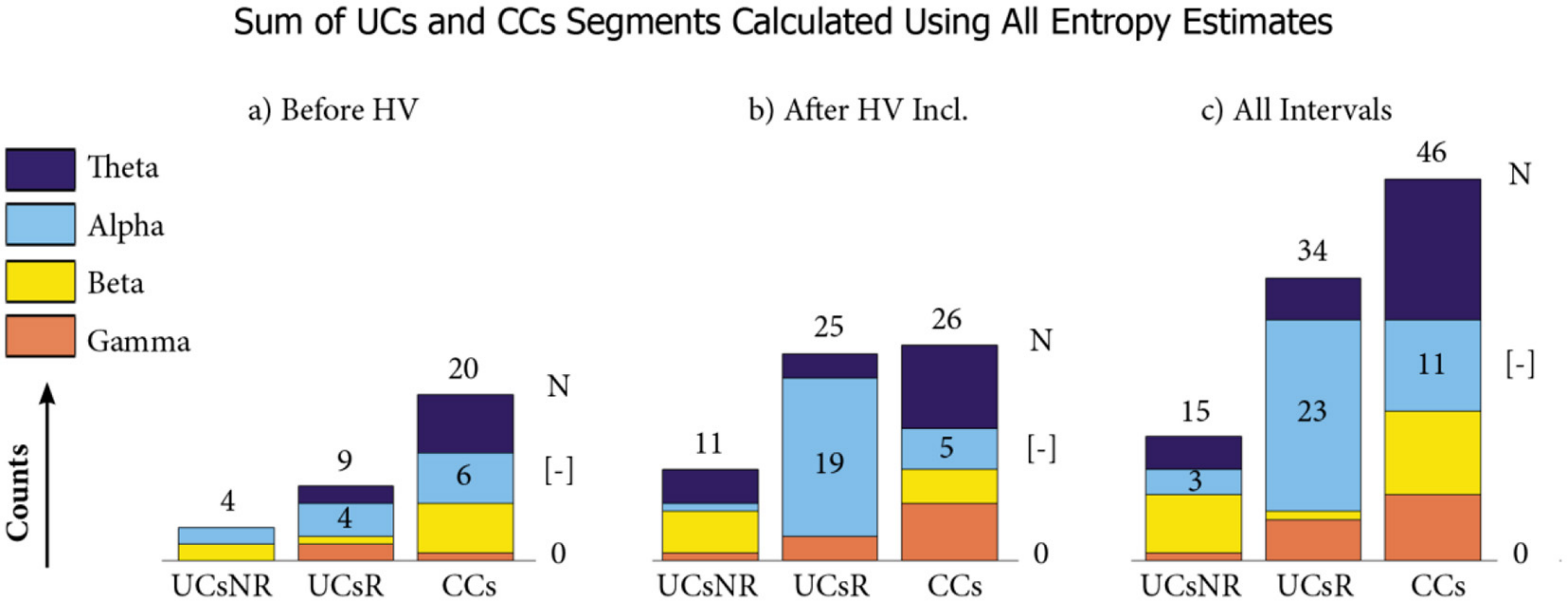
The distribution of UCs and CCs over the individual frequency bands. The number of unique characteristics of non-responders (UCsNR) is compared to the number of unique characteristics of responders (UCsR) and the number of common characteristics of responders and non-responders (CCs) summed across all Entropy estimators. Three different temporal views are shown: **(a)** counts before hyperventilation (including photic stimulation), **(b)** counts after photic stimulation (including hyperventilation), and **(c)** counts during all time intervals.

When splitting these numbers to the R and NR epileptic patient groups, and considering for simplicity the overall numbers, summed over all individual Entropy estimators, we observe that the VNS responders exhibit a higher number of segments (*N* = 80), differentiating them from HCs than the VNS non-responders (*N* = 61). Below, we specify which of these are unique (UC) for each individual R or NR group, and which are common characteristics (CC) of the epileptic patients. The distribution of UCs and CCs over the individual frequency bands is summarized in Figure 4.

**UCsR of VNS responders:** We identified *N* = 34 UCsR of VNS responders, as seen in Figure 4, column c). The alpha frequency band is essential here: *N* = 23 UCsR out of *N* = 34 UCsR were present in the alpha band. Focusing on the temporal distribution of UCsR in the alpha band, only one of the UCsR is found before PS, while they start to appear during the PS interval (*N* = 3). However, the most crucial time interval is the HV (see Figure 4, column b), during which numerous UCsR appear in most of the Entropy estimators (see Figure 4 for details). Specifically, we can identify *N* = 5 UCsR in alpha (out of 6 UCsR during HV across all frequencies). Their high prevalence persisted up to the end of the EEG recording, where in particular the last two time intervals, Rest-3 and Rest-4, contained in the alpha band N = 6 UCsR (7 UCsR across all frequencies) and *N* = 7 UCsR (9 UCsR across all frequencies) in sum for all Entropy estimators, respectively.

The remaining *N* = 11 UCsR of the VNS responders were found in the remaining frequency bands (*N* = 5 in theta, *N* = 1 in beta, and *N* = 5 in gamma). Compared to the alpha frequency band, the temporal distribution of UCsR in the other bands is more random (*N* = 5 UCsR appear in the first three intervals, while *N* = 6 UCsR appear during or after HV).

**UCsNR of VNS non-responders:** We identified *N* = 15 UCsNR of VNS non-responders, as seen in Figure 4, column a). Unlike UCsRs, the UCsNRs are bound mainly to the beta (*N* = 7) and theta (*N* = 4) frequency bands, while relatively fewer UCsNR segments are found in the alpha (*N* = 3) and gamma (*N* = 1) frequency bands. When focusing on UCsNR in the beta and theta frequency bands, we can see their temporal binding to HV, namely, *N* = 7 out of 11 UCsNR appear during the HV time segments.

**CCs of VNS responders and non-responders:** We found a total of *N* = 46 CCs that differentiated the R and NR epileptic patients from the HCs. The CCs occur mainly in the theta (*N* = 17 CCs) and alpha (*N* = 11 CCs) frequency bands.

When focusing on the temporal distribution of CCs in the theta and alpha bands, we see a pronounced bound with the intervals of the eyes opening/closure (OC-1 and OC-2): The OC-1 and OC-2 summed together display *N* = 9 and 6 CCs, respectively (out of the total *N* = 17, 11 CCs). This result is similar to the results of the CCs analysis using the RPW (Section 3.2).

**Summary of the Entropy analysis:** The VNS responders differ from the HCs in more UCs than the VNS non-responders. The responders differ from HCs predominantly in the alpha frequency band and temporally in the segments of stimulation by hyperventilation (and the subsequent rest segments with transient relaxation from the stimulation periods). For non-responders, the response to HV is also a crucial characteristic (Sklenarova et al., 2023), but unlike in responders, it is not found in alpha, but shifted to beta and gamma frequency bands. Both VNS responders and VNS non-responders exhibit abnormal or pathological reactions to eye opening, predominantly in the alpha and theta frequency bands. All these findings are presented in Figures 3, 4 with details corresponding to individual Entropy estimators given in their respective panels. Figures in the supplement represent the distribution of relative Entropy values across the scalps for each Entropy estimator (these are analogous to Figure 2a), showing the results of the RPW analysis.

### 3.4 Results summarization

**Figure 5:**
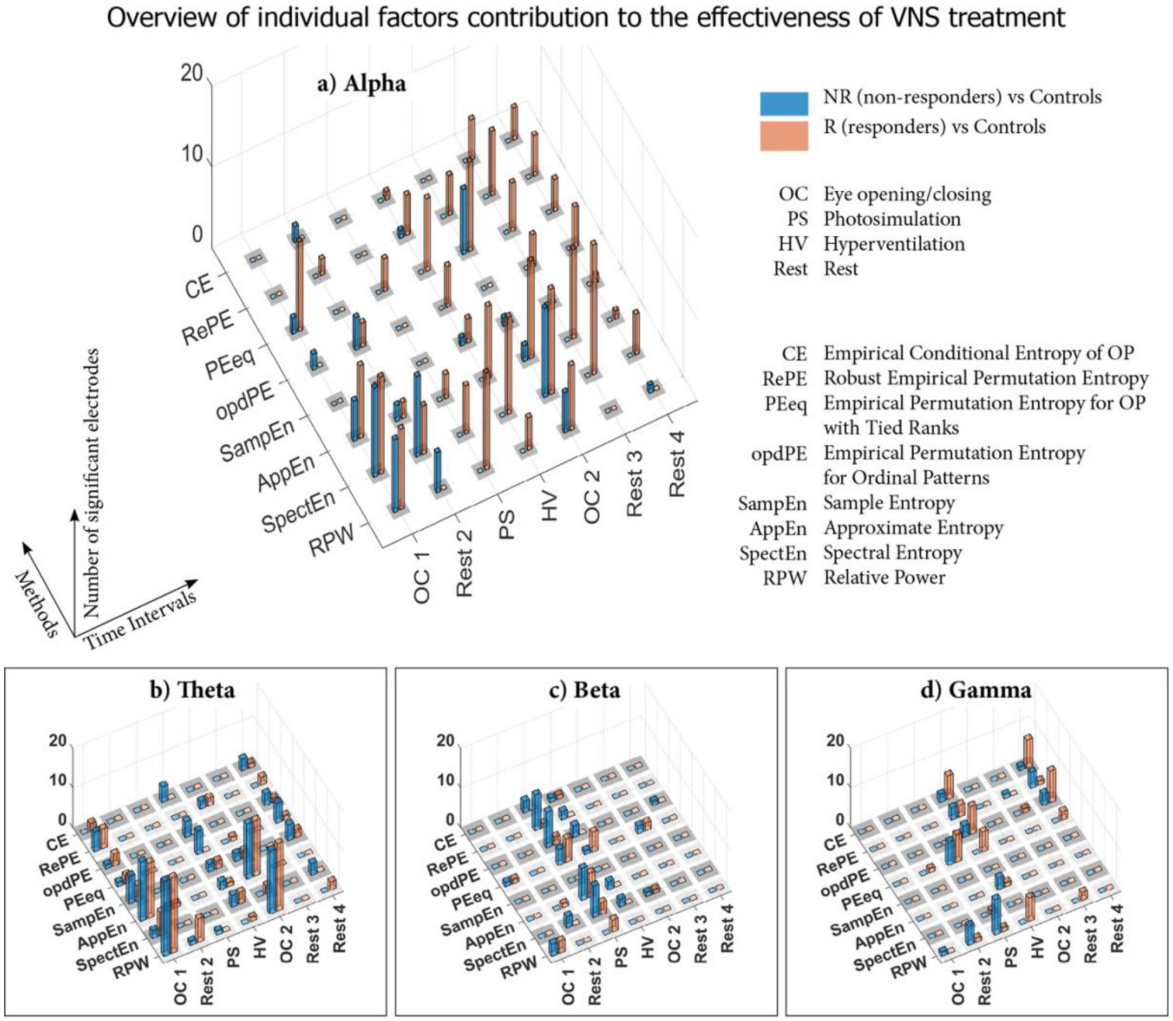
Results summarization–Relative Power (RPW) and Entropy. Overview of the numbers of electrodes with statistically significant differences between responders (R) vs. Healthy Controls (HC), non-responders (NR) vs. HC subject groups, based on all considered measures: RPW and all Entropies, in all eight time intervals corresponding to different stimulations and rest stages, see Figures 2 and 3. The individual results for RPWs and each Entropy estimator follow (RPW–Relative power, SpecEn-Spectral Entropy, AppEn–Approximate Entropy, SampEn–Sample Entropy, PEeq–Empirical Permutation Entropy for Ordinal Patterns with Tied Ranks, opdPE–Empirical Permutation Entropy for Ordinal Patterns, RePE–Robust Empirical Permutation Entropy, H–Conditional Entropy). **(A)** Alpha frequency range; **(B)** Theta frequency range; **(C)** Beta frequency range; **(D)** Gamma frequency range.

The results, analyzed from various detailed perspectives in the previous section, are summarized in Figure 5. This synoptic view highlights the markedly different alpha frequency band in comparison to the remaining three bands, as well as the importance of PS and HV.

## 4 Discussion

Based on our group's previous results, it appears that both the basic RPW and the more sophisticated Entropy methods reflect the pathophysiological mechanisms of epilepsy and the mechanisms of VNS action, leading to their successful application as features that differentiate between VNS responders and non-responders (Chase et al., 1967; Sangare et al., 2020).

In the present manuscript, we focused on the electrophysiological differences between VNS responders vs. HC and VNS non-responders vs. HC. We analyzed various EEG features using two approaches: RPW and several Entropy measures in pre-implantation, including stimulation via PS and HV.

Focusing on the summarization of our results, even a brief look indicates that the responses captured by all the methods are markedly different in the alpha frequency band in comparison to the remaining three bands: The patterns observed in the theta, beta and gamma bands are relatively more straightforward compared to alpha and (i) contain predominantly the CCs differentiating the epileptic patients from the HCs and (ii) are relatively highly organized in the temporal sense, related to different types of stimulation and rest time intervals. In sharp contrast, the results in the alpha frequency band display a large number of UCs, predominantly for the VNS responder epileptic patient group. The occurrence of these UCsR starts temporally with the time segment of the PS and even more markedly in the following HV segment. Notice that, in addition to the sole numbers of UCsR, the particular numbers of significant electrodes are also large in many cases, indicating a possibly global character of neuronal dynamics, which differentiates the VNS responders from the HC group.

On the contrary, the UCsNR exhibit a different pattern. They are not bound to the alpha frequency; their distribution among other frequency bands is more random, however, their binding to the beta frequency predominates. Also, UCsNR have a different time distribution compared to UCsR.

From this perspective, the interpretation of the significance of PS and HV as standard activation methods, and the role of alpha appears to be crucial.

PS and HV are activation methods routinely used in the investigation of epileptic patients. From the clinical point of view, both increase the brain excitability and the probability of positive EEG findings. Based on these “clinical reasons,” the attempts for their application in clinical research seem to be logical.

When focusing on its pathophysiological nature, hyperventilation is believed to induce seizures through hypocapnia and vasoconstriction (Salvati and Beenhakker, 2019; Milan et al., 2024). Seyal et al. (1998) proposed that hyperventilation-induced increases in excitability may contribute to clinical phenomena such as the facilitation of spike-wave discharges. Interesting results in the context of our research were published by Mazzucchi et al. (2017). The authors investigated the effect of HV on brain functional connectivity and estimated the differences in response to HV between patients with epilepsy and healthy controls. They found that HV is associated with the activation of the cingulate cortex and modifies brain connectivity. The activation method resulted in distinct brain connectivity patterns in patients with epilepsy and healthy subjects. The epileptic patients were characterized by significantly increased connectivity in alpha in bilateral anterior cingulate cortex, subcallosal gyrus, medial frontal gyrus, and rectal gyrus (alpha frequency range), and in gamma in posterior cingulate cortex. In the control group, there was an increase in connectivity in the alpha band in the left inferior frontal gyrus and insula, in the beta band in the anterior cingulate cortex, insula, parahippocampal gyrus, inferior frontal gyrus, and subcallosal gyrus, and in the gamma band in the posterior cingulate gyrus and precuneus.

Less is known about photic stimulation and its pathophysiological connotations. It is postulated that flashing lights induce hyperexcitability in the visual cortex, leading to abnormal electrical activity and seizures (Kasteleijn-Nolst Trenité et al., 1999). In literature, “pathological” states characterized by the alteration of brain connectivity present in response to visual stimulation were described in different contexts; we can name the studies focusing on schizophrenia (Galdino et al., 2022), Alzheimer's diseases (Wada et al., 1998), migraine with and without aura, (de Tommaso et al., 2013) or autistic spectrum disorder (Lazarev et al., 2010).

Based on these findings, it seems that epilepsy as a disease leads to the alteration of brain functions, which Entropy measures and RPW analysis can reflect.

The interpretation of alpha frequency distinctions is linked to the inhibition of the cerebral cortex. Jensen and Mazaheri (2010) demonstrated that alpha activity is responsible for pulsed inhibition, thereby reducing the processing capabilities of a given area (Jensen and Mazaheri, 2010). On the other hand, active processing within the cerebral cortex is reflected by synchronization in the gamma band, accompanied by a concomitant decrease in alpha. In the context of epilepsy, the alpha band activity is hypothesized to serve as an endogenous mechanism that opposes the pathophysiological hallmark of seizures–the aberrant, large-scale neuronal synchronization. By promoting a relatively desynchronized state, alpha activity may play a crucial role in suppressing or resisting seizure-related hypersynchrony.

Ultimately, we would like to discuss and interpret the clinical significance of CCs. We speculate that CCs are mainly connected to the particular degree of encephalopathy, which could be found in most epileptic patients implanted with VNS. This encephalopathy is conditioned by several factors: underlying causes of epilepsy, ongoing seizures, and the effect of ASM. The CCs are bound to the episodes of eye-opening. In healthy subjects, episodes of eye opening are associated with a decrease in alpha frequency activity, which is replaced by beta frequency activity. This phenomenon was initially described by Berger and is known as Berger's reaction (Berger, 1969). We speculate that the changes observed in RPW and Entropy in both VNS responders and non-responders primarily reflect this abnormal reaction and could serve as an indicator of brain dysfunction.

The retrospective design of our study sets certain limitations which could be overcome in subsequent prospective studies: In particular, the data recorded at relatively low sampling frequency (max. 128 Hz) specifically precluded a robust investigation of the high-gamma band, focusing our results on activity within the alpha and lower gamma ranges (Dolezalova et al., 2022).

## 5 Conclusion

We analyzed electrophysiological characteristics for three subject groups to better understand the differences between the two groups of epilepsy patients (R and NR) treated with VNS and healthy control (HC) subjects. The observed differences were characterized by applying two complementary analytical approaches to the scalp EEG data. Specifically, we computed the (i) relative mean power (RPW) and (ii) different information entropy estimators. Responders to VNS therapy exhibit a distinct neurophysiological profile, with significant deviations from healthy controls (HCs) that are localized both spectrally and temporally. Specifically, these differences are most pronounced in the alpha frequency band and manifest primarily during and immediately following a hyperventilation stimulation.

In contrast, VNS non-responders display a different pattern of difference from HCs: While the time segment of hyperventilation persists to be crucial, the spectral locus of significant differences between VNS non-responders and HCs is shifted from the alpha frequency band to the beta and theta bands. We also evaluated common characteristics (CCs), differentiating the epileptic patients from HCs. These CCs are likely linked to some degree of encephalopathy and are present primarily in alpha and theta frequency ranges during time intervals associated with eye opening and subsequent closure.

We believe that these findings can be helpful in clinical practice and inform further research into the mechanisms underlying the action of VNS therapy. The characteristics of VNS responders are their increased reactivity to external stimuli. While our experimental design explicitly employed Photic Stimulation (PS) and Hyperventilation (HV) as external stimuli, we suggest that the VNS can act as a different additional source of a sustained background neurostimulation that fundamentally shapes the observed distinct electrophysiological response of the responder group subjects. In other words, we hypothesize that the stimulations by HV, PS, and crucially also the VNS, may trigger distinct neurological response due to a common (though yet unknown) underlying mechanism specific to the responder group subjects.

Our findings suggest that the VNS responder group exhibits increased susceptibility relative to both healthy subjects and non-responders. While the specific neurological mechanisms underlying these observations remain unknown, we believe that our study may inspire further research in this area. Despite the current lack of detailed interpretations regarding these mechanisms, we posit that the RPW and entropy estimators utilized in our analysis could already serve as clinically relevant indicators for considering VNS therapy.

The highest sensitivity of alpha may be related to its inhibitory impact on the cerebral cortex and can be linked to the reduction of epileptogenic potential. We can speculate that this lower epileptogenic potential is responsible for the seizure reduction observed in VNS responders. These results can be interpreted as a specific pattern of VNS responders, and bind their characteristics to activation methods in alpha, differentiating them from both VNS non-responders and HCs.

## Data Availability

The data analyzed in this study is subject to the following licenses/restrictions: The dataset can be made available upon an reasonable request. Requests to access these datasets should be directed to chladek@isibrno.
