# Supplementary material for: Complex network behavior in epileptic patients treated with Vagus Nerve Stimulation (VNS): VNS responders exhibit a unique pattern, different from VNS non-responders and healthy controls
Source: Front Neurosci. 2026 Jan 27;19:1662068. doi: 10.3389/fnins.2025.1662068 (PMC12886412; doi:10.3389/fnins.2025.1662068)

**Supplementary Materials:**

**Supplementary Material 1: Entropy analysis – differences between VNS responders or VNS non-responders and healthy controls (HCs)**

*The x axis represents eight consecutive time intervals (Rest-1, OC-1, Rest-2, PS, HV, OC-2, Rest-3, and Rest-4). The y axis displays the results of individual Entropy methods, which were separately calculated for VNS responders vs. HCs and VNS non-responders vs HCs across individual frequency bands (theta, alpha, beta, and gamma). Each head represents the Entropy method applied to the given EEG segment, characterized by frequency and time interval, with entropy values calculated separately for each scalp electrode. White dots indicate only those electrodes where significant differences were found between responders/non-responders and HCs vs at the p≤0.05 significance level.*

- *Spectral Entropy*
- *Approximate Entropy*
- *Sample Entropy*
- *Empirical Permutation Entropy for Ordinal Patterns*
- *Empirical Permutation Entropy for Ordinal Patterns with Tied Ranks*
- *Robust Empirical Permutation Entropy*
- *Conditional Entropy*

1. *Spectral Entropy (SpectEn)*


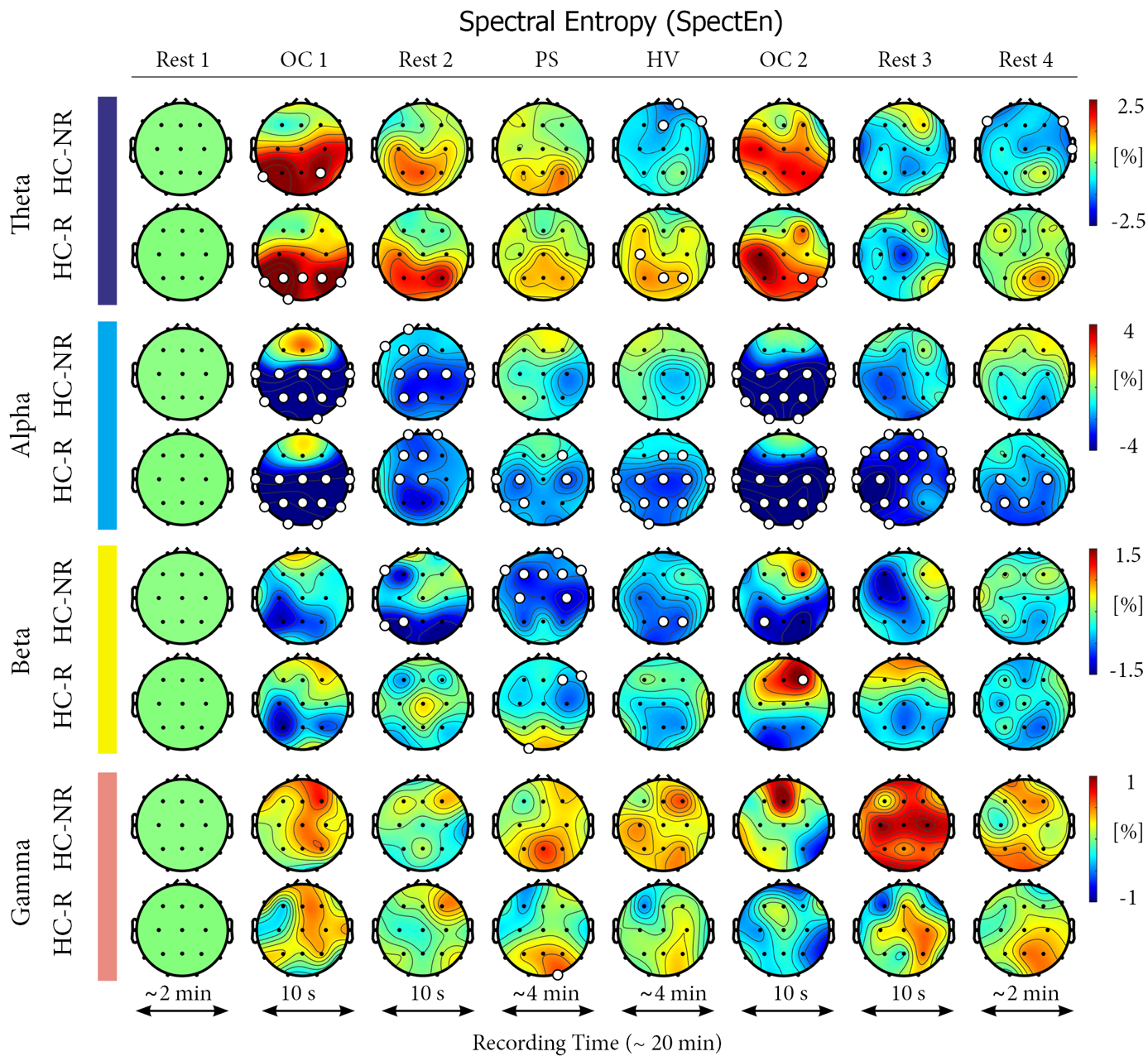


1. *Approximate Entropy (AppEn)*


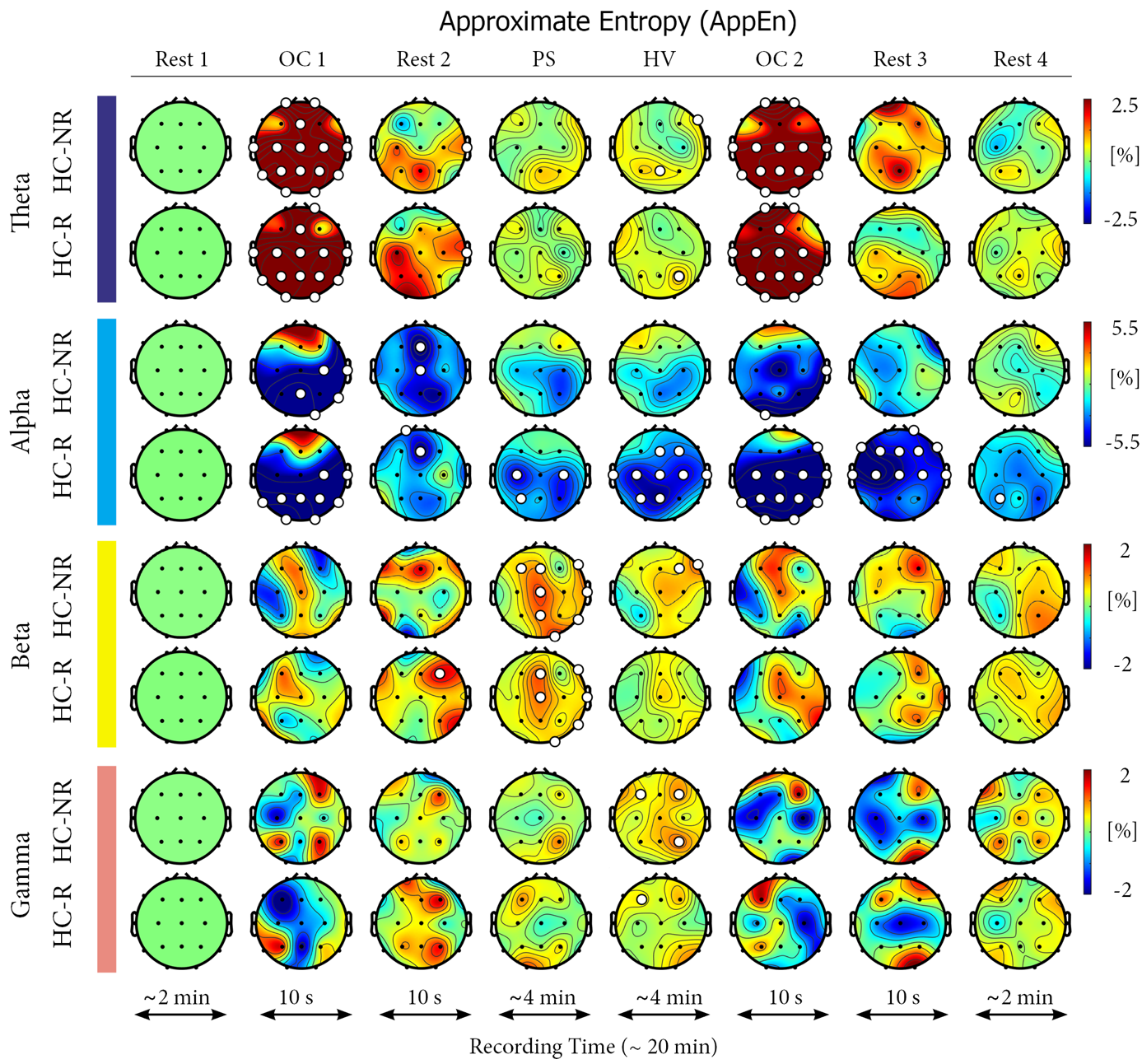


1. *Sample Entropy (SampEn)*


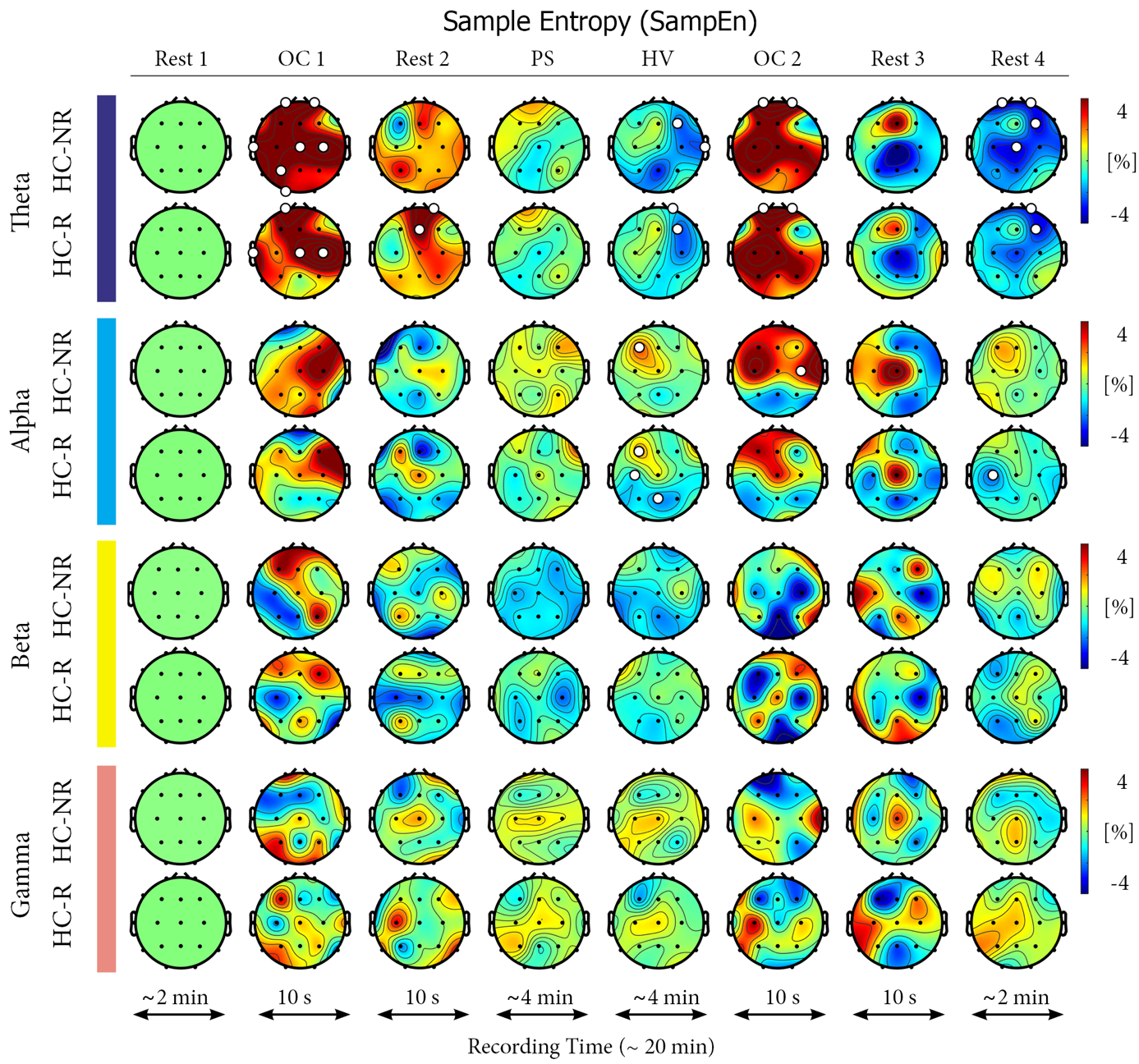


1. *Empirical Permutation Entropy for Ordinal Patterns with Tied Ranks (PEeq)*


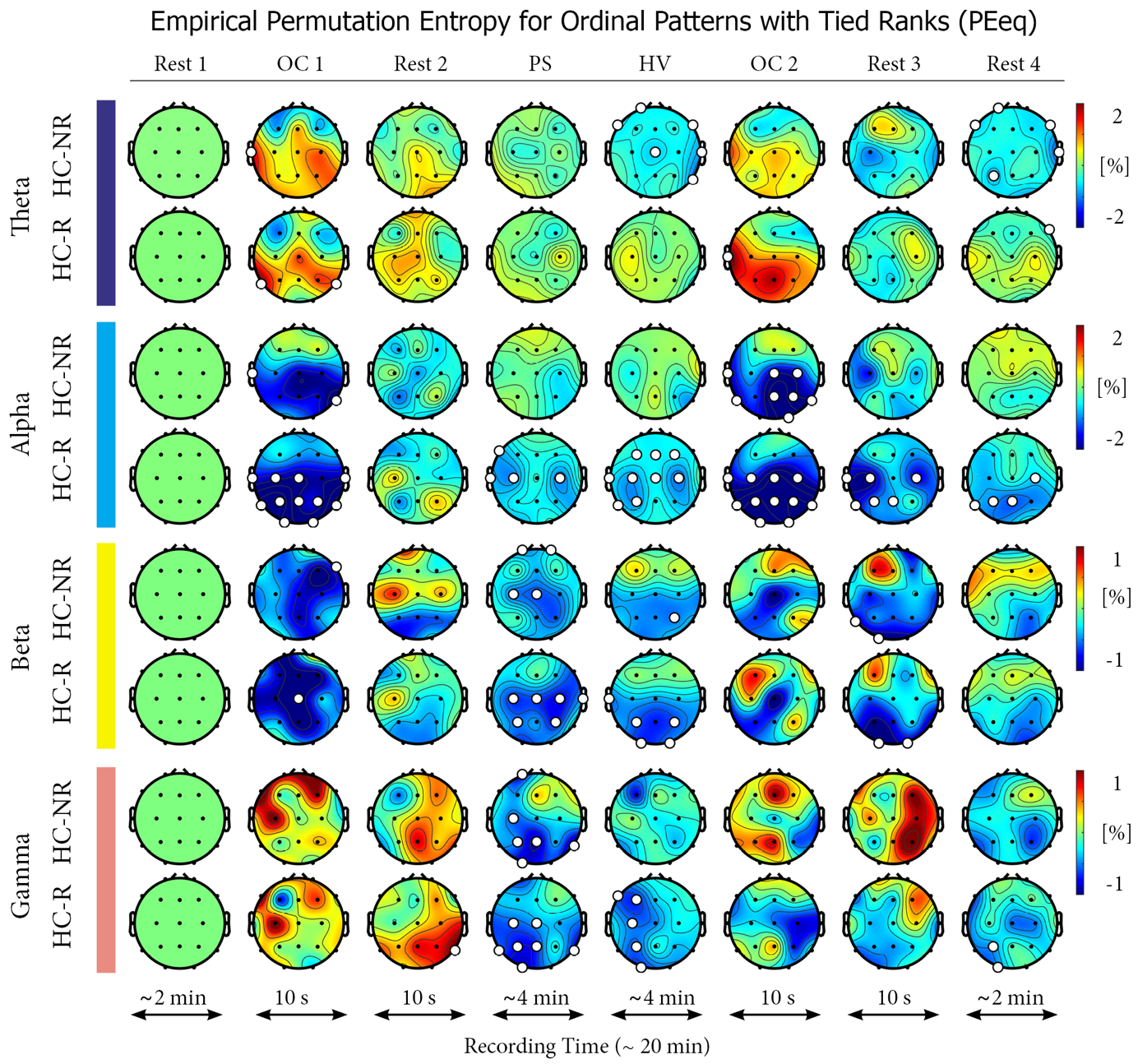


1. *Empirical Permutation Entropy for Ordinal Patterns (opdPEn)*


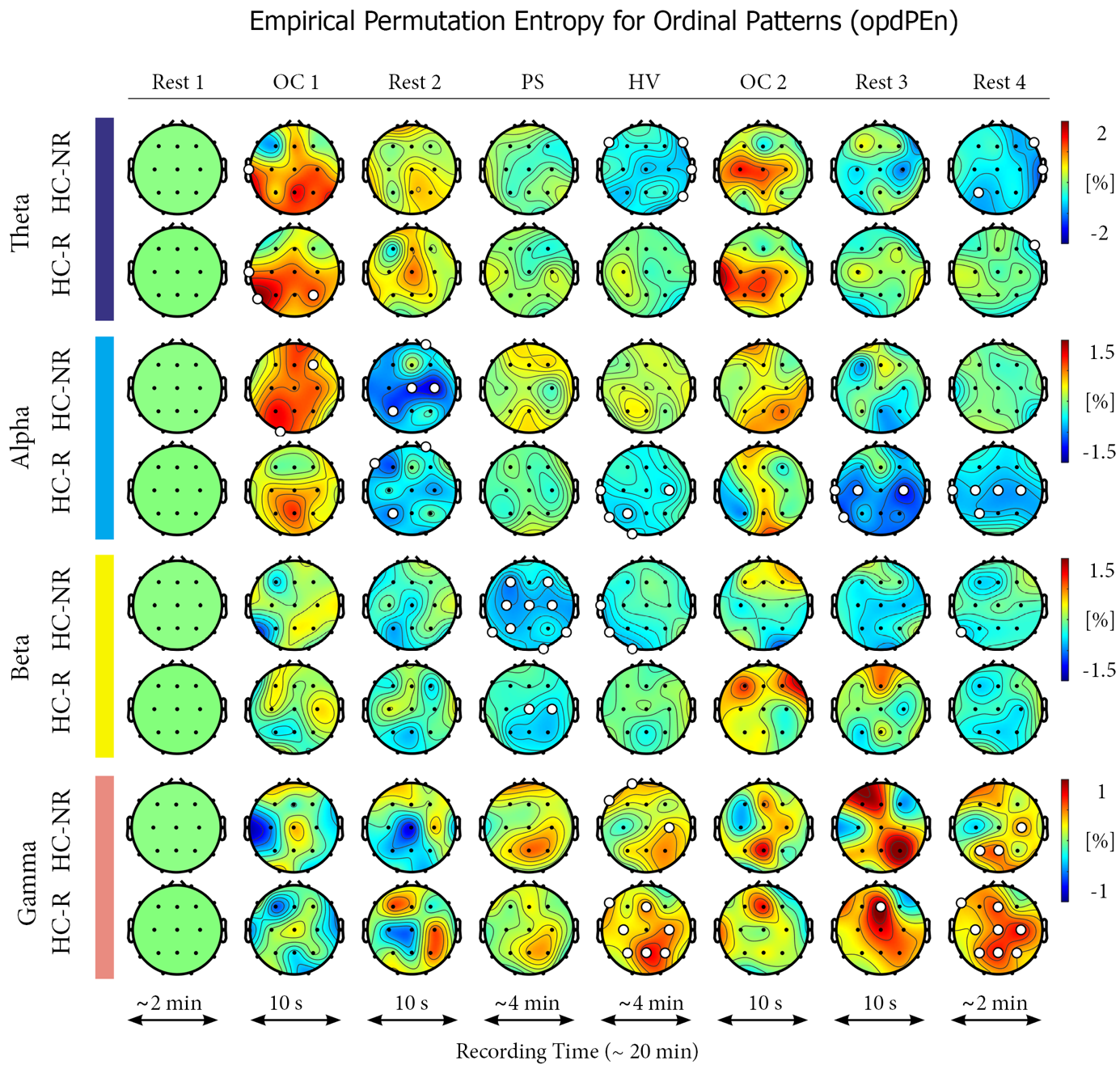


1. *Robust Empirical Permutation Entropy (RePE)*


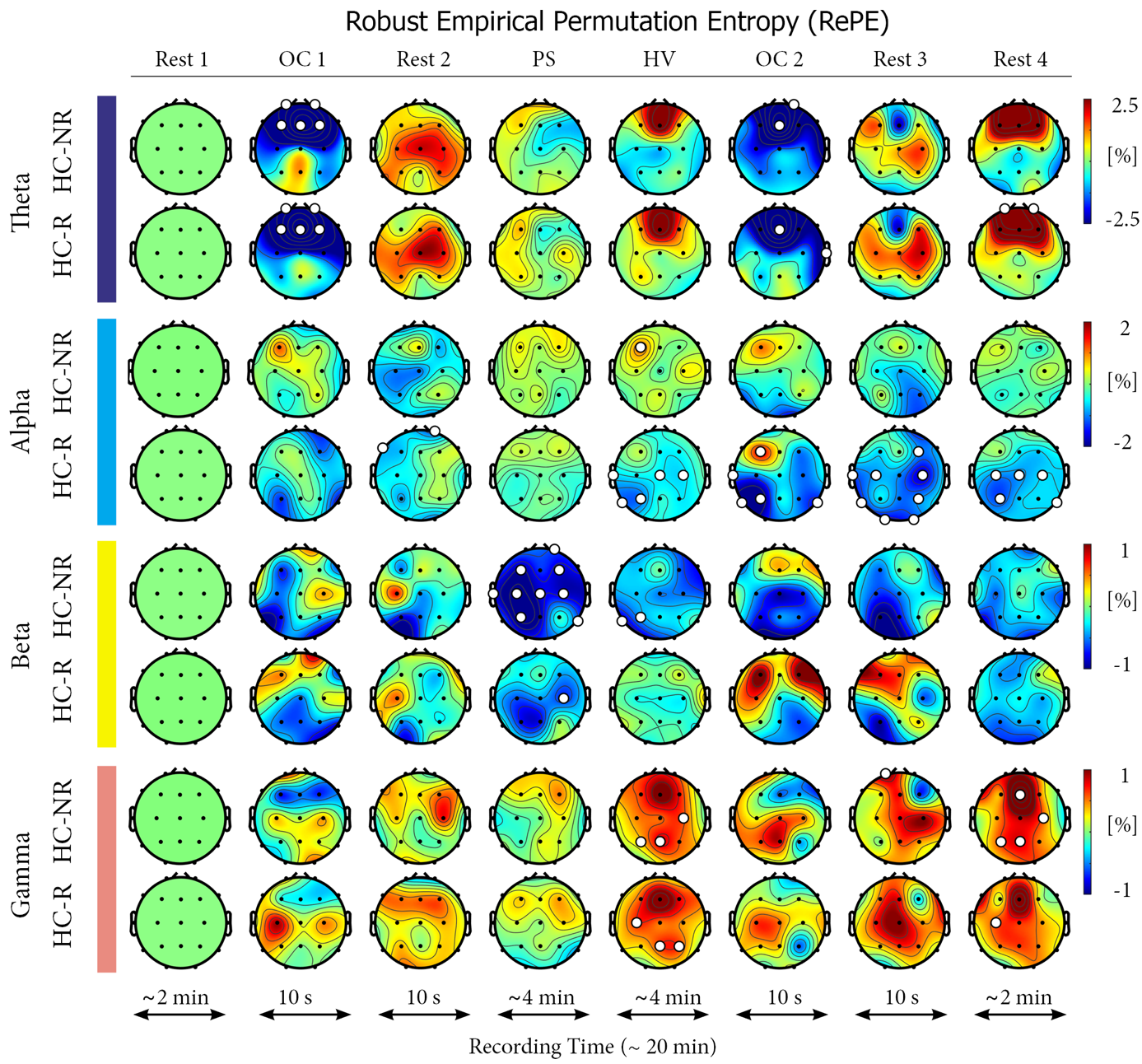


1. *Conditional Entropy (CE)*


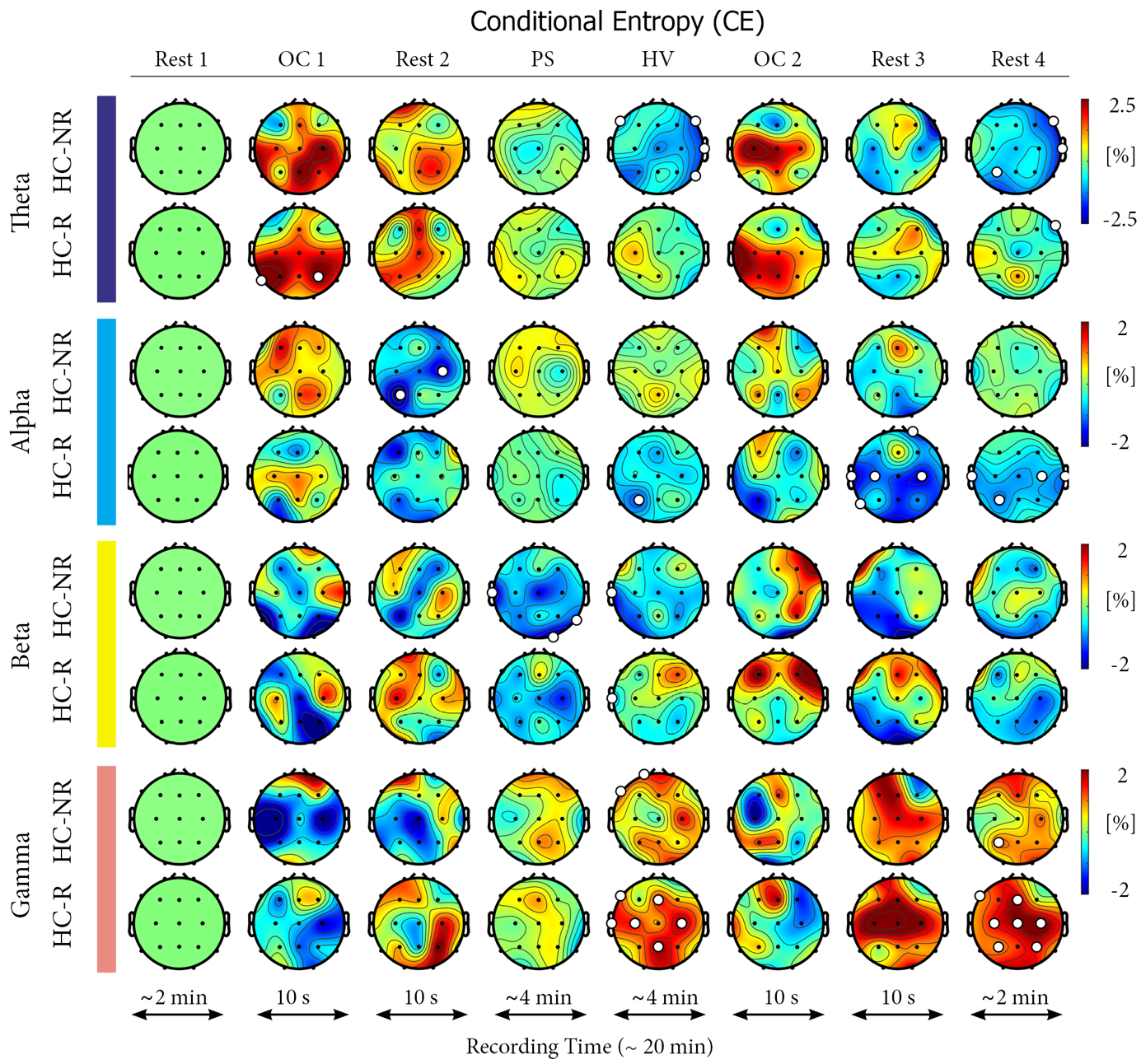

Supplement: Supplementary file 1 [file Supplementary_file_1.docx]
